# Plasmonic magnesium nanoparticles decorated with palladium catalyze thermal and light-driven hydrogenation of acetylene†

**DOI:** 10.1039/d3nr00745f

**Published:** 2023-03-29

**Authors:** Vladimir Lomonosov, Thomas M. R. Wayman, Elizabeth R. Hopper, Yurii P. Ivanov, Giorgio Divitini, Emilie Ringe

**Affiliations:** a Department of Materials Science and Metallurgy, University of Cambridge 27 Charles Babbage Road Cambridge CB3 0FS UK er407@cam.ac.uk +44 (0)1223 334567 +44 (0)1223 334330; b Department of Earth Sciences, University of Cambridge Downing Street Cambridge CB2 3EQ UK; c Department of Chemical Engineering and Biotechnology, University of Cambridge Philippa Fawcett Drive Cambridge CB3 0AS UK; d Electron Spectroscopy and Nanoscopy, Istituto Italiano di Tecnologia Via Morego 30 16163 Genova Italy

## Abstract

Bimetallic Pd–Mg nanoparticles were synthesized by partial galvanic replacement of plasmonic Mg nanoparticles, and their catalytic and photocatalytic properties in selective hydrogenation of acetylene have been investigated. Electron probe studies confirm that the Mg–Pd structures mainly consist of metallic Mg and sustain several localized plasmon resonances across a broad wavelength range. We demonstrate that, even without light excitation, the Pd–Mg nanostructures exhibit an excellent catalytic activity with selectivity to ethylene of 55% at 100% acetylene conversion achieved at 60 °C. With laser excitation at room temperature over a range of intensities and wavelengths, the initial reaction rate increased up to 40 times with respect to dark conditions and a 2-fold decrease of the apparent activation energy was observed. A significant wavelength-dependent change in hydrogenation kinetics strongly supports a catalytic behavior affected by plasmon excitation. This report of coupling between Mg's plasmonic and Pd's catalytic properties paves the way for sustainable catalytic structures for challenging, industrially relevant selective hydrogenation processes.

## Abstract 2

Plasmonic Mg cores capture light to decrease the activation energy and increase the rate of acetylene hydrogenation on Pd nanoparticles.

## Graphical abstract

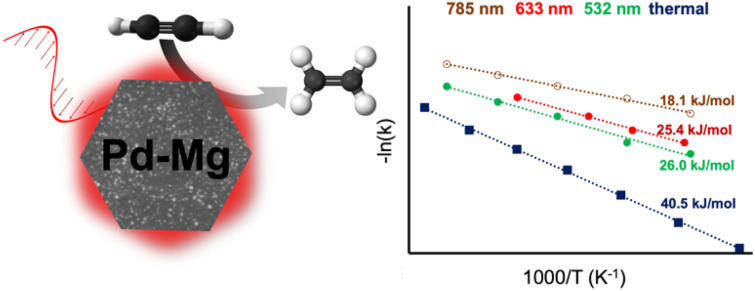

## Introduction

Metal nanoparticles (NPs) are central to heterogeneous catalysis research owing to the unique properties of nanomaterials. Their substantially higher surface-to-volume ratio than that of bulk materials provides a larger number of active sites per volume, and potentially different sites such as edges and corners, resulting in enhanced catalytic performance. The ability of some metals to sustain oscillations of their free electron density called localized surface plasmon resonances (LSPRs), make them especially attractive to catalysis research.^1–3^ LSPRs generate strongly enhanced electromagnetic fields near the NP surface that enhance scattering signals and have been used to spectroscopically track chemical reactions occurring on surfaces.^4,5^ Furthermore, the decay of LSPRs generates a sequence of energetic products, from excited charge carriers^6–8^ to, eventually, localized heat.^9–11^ Numerous studies demonstrated that both these plasmon decay products are capable of activating chemical transformations by providing energy to molecules adsorbed on the NP's surface.^12–19^ A notable architecture for plasmon-enhanced catalysis is the antenna–reactor system,^12,18^ where the plasmonic core “antenna” couples light into the catalytic “reactor”, often small particles decorating the central plasmonic structure. This is particularly attractive for the coupling of a cheap plasmonic core with a rare catalytic metal, as the latter is needed in quantities one to two orders of magnitude smaller than the plasmonic metal. Indeed, heterostructures based for example on plasmonic Al NPs have demonstrated enhanced photocatalytic performance in multiple reactions including methylene blue^20,21^ and Rhodamine B^22^ decomposition, selective acetylene hydrogenation,^12^ carbon dioxide reduction,^23^ nitrous oxide decomposition,^24^ carbon–fluorine bond activation,^25^ and methane dry reforming.^26^

Mg is an alternative earth-abundant plasmonic metal attracting growing attention owing to its ability to sustain LSPRs across the ultraviolet, visible, and near-infrared wavelengths,^27–31^ and its superior resonance quality factor to Al across most of this energy range.^32^ Partial galvanic replacement of Mg with Au, Ag, Fe, and Pd has been shown to create bimetallic, decorated nanostructures.^33^ These structures offer an opportunity to investigate the coupling of Mg's plasmonic properties with well-established catalytically active materials for photocatalysis applications.

The model reaction we use, selective hydrogenation of acetylene, is also a challenging and industrially relevant type of chemical transformation. Ethylene is one of the main building blocks of basic organic chemistry and petrochemical synthesis and is produced by thermal and catalytic dehydrogenation and cracking of C_2+_ hydrocarbons. The resulting ethylene usually contains other hydrocarbons including acetylene which is further removed by selective catalytic hydrogenation to obtain polymer grade C_2_H_4_. We use Mg NPs decorated with Pd as a model catalytic system since supported Pd-based catalysts have been widely used in the chemical industry for hydrogenation reactions.^34,35^ However, in the case of hydrogenation of acetylene, monometallic Pd suffers from low selectivity to ethylene due to overhydrogenation. Meanwhile, coupling of Pd with other metals allows to improve selectivity substantially.^36–48^ For example, the addition of Ag,^36–41,49,50^ Au,^39^ Cu,^46^ Zn,^47^ In,^48^ Ga,^42,43^ Sn^44,51^ to Pd can suppress overhydrogenation and therefore increase the selectivity to ethylene.

Here we demonstrate that coupling of finely dispersed Pd with Mg core allows to achieve a substantially higher selectivity to ethylene compared to the conventional monometallic Pd catalysts.^47,48,52^ Particularly, we obtain a stable ethylene selectivity of more than 50% at the complete conversion of acetylene at 60 °C. In addition to the improved thermally driven catalytic performance, the Pd–Mg nanostructures exhibit an excellent photocatalytic performance under laser beam excitation at room temperature. Our results show that initial hydrogenation rate increased up to 40 times with respect to dark conditions and was accompanied by a 2-fold decrease in the apparent activation energy. Although photothermal heating inevitably contributes to the activation of the reagents, the wavelength-dependent alteration in hydrogenation kinetics under light illumination, revealed by initial rates kinetic analysis, rules out solely photothermal activation and strongly suggests a catalytic behavior enhanced by plasmon excitation. Such photocatalysts based on earth-abundant Mg cores pave the way for large scale, cost-effective catalysts that can interact with the entire solar emission range for sustainable plasmon-enhanced catalysis.

## Experimental section

### Materials

Lithium pellets (99%), naphthalene (99%), 1.0 M di-*n*-butylmagnesium (MgBu_2_) in heptane, anhydrous tetrahydrofuran (THF), anhydrous isopropanol (IPA), polyvinylpyrrolidone (PVP) (average mol. weight 10 000), Na_2_PdCl_4_ (99.99%), silica gel (high-purity, 60 Å, 60–100 mesh), and glass spheres (9–13 μm) were purchased from Sigma-Aldrich. Poly(styrene-*b*-4-vinyl pyridine) (PS-P4VP) was purchased from Polymer Source, Inc. A gas mixture containing 5 vol% of acetylene in nitrogen was purchased from BOC.

### Catalyst synthesis

Mg NPs were synthesized by the reduction of di-*n*-butylmagnesium, as previously reported.^30^ For a typical reaction, 0.028 g of lithium (4.05 mmol), 0.530 g of naphthalene (4.14 mmol), 0.020 g PVP and 10.75 mL of anhydrous THF were added to a 25 mL Schlenk flask under an Ar atmosphere and sonicated for 1 h, producing a dark green lithium naphthalenide (LiNapht) solution. 1.75 mL of MgBu_2_ in heptane (1.0 M, 1.75 mmol) was injected into lithium naphthalenide under vigorous stirring and left to react overnight at room temperature (20 °C) before quenching the reaction mixture with anhydrous IPA. The solid product was recovered by centrifugation and then cleaned by repeated centrifugation and redispersion steps in anhydrous THF twice and anhydrous IPA twice, before redispersing in anhydrous IPA. All glassware was washed with aqua regia (1 : 3 HNO_3_ : HCl) and flame-dried under a vacuum before use.

Decoration of Mg NPs with Pd was performed using an optimized protocol previously reported by Asselin *et al.*^33^ In a typical decoration experiment, 1 mL of the Mg NP suspension was diluted with 2 mL of anhydrous IPA before the injection of 3 mL of Na_2_PdCl_4_ solution in anhydrous IPA. The Mg content of as-prepared Mg sample was determined by inductively coupled plasma mass spectrometry (ICP-MS) and was used to calculate the stoichiometric amount of Na_2_PdCl_4_. The mixture of Mg NPs with Pd precursor was left to react for 1 h in a sealed vial under stirring. The Pd–Mg bimetallic nanostructures were recovered by centrifugation and residual byproducts were removed by repeated, generally three times, centrifugation and redispersion in anhydrous IPA.

Pd and Pd–Mg supported on SiO_2_ were prepared by incipient wetness impregnation of preliminary dried silica gel (120 °C overnight). For Pd–Mg/SiO_2_ samples, 20 mg of silica gel was impregnated with 1.2 mL of concentrated (1.9 mg mL^−1^) suspension of Pd–Mg NPs, and dried overnight in a desiccator under an Ar atmosphere. Pd/SiO_2_ samples were prepared by impregnation of 20 mg of SiO_2_ with 1.2 mL of Na_2_PdCl_4_ solution in IPA (concentration of Pd precursor was adjusted in accordance with the desired stoichiometry) followed by drying in air and reduction in a flow of H_2_ at 250 °C for 4 hours.

To enhance light penetration in photocatalysis experiments (results from Fig. 3 onwards), Pd–Mg NPs were supported on glass spheres instead of SiO_2_. First, glass spheres were immersed in piranha solution (3 : 1 H_2_SO_4_ : H_2_O_2_) at ∼80 °C for 1 hour and then cleaned by repeated centrifugation and redispersion steps in DI water to neutral pH followed by one additional step in THF. After the cleaning, the glass spheres were immersed in 0.05 mg mL^−1^ of PS-P4VP solution in THF for 5 min, cleaned with THF two times and dried in a Petri dish in air at room temperature. To obtain Pd–Mg supported samples, 10 mg of block copolymer-covered glass spheres were immersed in 0.5 mL of concentrated (1.6 mg mL^−1^) suspension of Pd–Mg NPs and left for one hour with a brief vortexing of the sample every 15 min. The supported Pd–Mg NPs were recovered by centrifugation and dried in a desiccator under an Ar atmosphere.

### Catalyst characterization

**Fig. 1:**
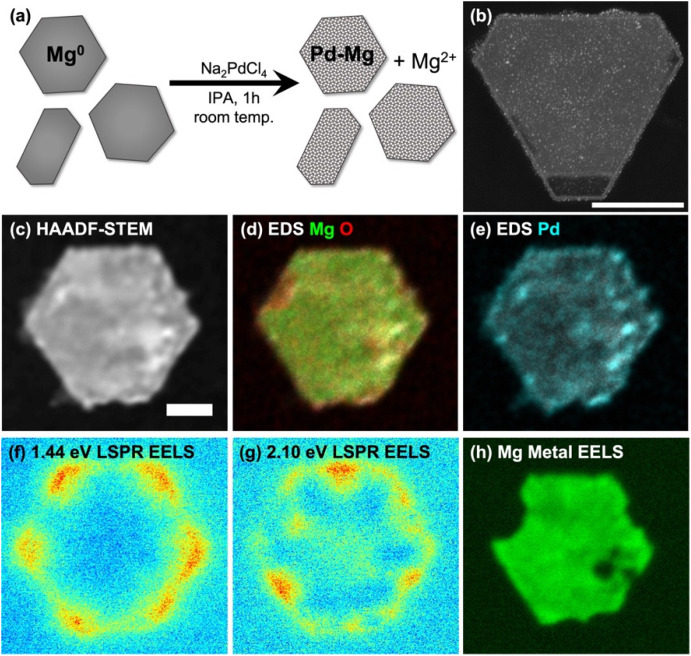
Partial galvanic replacement of Mg NPs with Na_2_PdCl_4_ produces Pd-decorated Mg NPs that retain their metallic and plasmonic character. (a) Schematic of the galvanic replacement, (b) HAADF-STEM of a single crystalline Mg NP decorated with 3 mol% Pd, (c) HAADF-STEM acquired concurrently to STEM-EELS, (d and e) STEM-EDS showing the spatial distribution of Mg, O and Pd, (f and g) STEM-EELS intensity integrated in a 0.105 eV window centred at 1.44 eV and 2.10 eV, showing the excitation of LPSRs, and (h) STEM-EELS intensity integrated in a 0.105 eV window centred at 10.425 eV showing the bulk metallic plasmon of Mg. Scale bars, 100 nm.

SEM imaging of samples drop-cast on Si wafers was performed on a Quanta-650F field emission gun scanning electron microscope (SEM), operated at 5 kV, and equipped with an Everhart–Thornley detector for secondary electron imaging. NPs were classified as hexagonal platelets if they had six sides of approximately equal length and rod-shaped if one dimension was elongated. The size of hexagonal platelets was defined as the distance between opposite corners; for rods, as the longest length. High angle annular dark field scanning transmission electron microscope (HAADF-STEM) images (Fig. 1(b) and S4, 5†) of samples drop cast on a Cu-supported lacey ultrathin carbon film were acquired at 200 kV on a FEI Osiris STEM. HAADF-STEM, STEM-energy dispersive X-ray spectroscopy (STEM-EDS) and STEM-electron energy loss spectroscopy (STEM-EELS, Fig. 1(c)–(h) and S6–9†) of samples drop cast on 20 nm thick Si_3_N_4_ membranes (SIMPore) were acquired on a monochromated ThermoFisher Spectra 300 operated at 60 kV using a Gatan spectrometer (GIF Continuum HR) with a dispersion 0.015 eV per channel and a corresponding zero-loss peak full width at half maximum of 0.08 eV. Data were acquired with a convergence angle of 1 mrad. Hyperspectral images consisted of an array of 1024 × 1024 pixels^2^, with an acquisition time of 0.01 ms per pixel. EDS spectrum imaging was performed with a Dual-X EDS detector with a collection solid angle of 1.7 sr.

ICP-MS was performed using a PerkinElmer NexION 2000-S mass spectrometer. Samples were digested in an aqueous matrix with 10 vol% of ultrapure nitric acid (maximum 10 parts per trillion metal traces) for at least 10 min before analysis. Optical extinction spectra were measured using a Thermo Scientific Evolution 220 spectrophotometer.

### Catalytic experiments

The catalytic experiments were carried out in a horizontally oriented packed bed reactor (Harrick Sci high-temperature reaction chamber) equipped with an electrical heater (24 V) and a thermocouple (K-type) located at the bottom of the sample well. The CaF_2_ window (6 mm) at the top of the reactor allowed both light irradiation of the catalyst surface and detection of its temperature with an IR camera (FLIR One Pro). The flow rates of C_2_H_2_ (5 vol% in N_2_), H_2_ (100%), and N_2_ (100%) were maintained by mass flow controllers (Bronkhorst HighTech). The reactants and product compositions were analyzed online by gas chromatography (GC) analysis (Thermo Scientific TRACE 1600) with thermal conductivity and flame ionization detectors. No other products of acetylene hydrogenation except ethylene and ethane were detected in these conditions. The process parameters, namely acetylene conversion *X*_C_2_H_2__ (%), ethylene selectivity *S*_C_2_H_4__ (%), and acetylene conversion rate *W*_C_2_H_2__ (mmol s^−1^ g^−1^), were calculated based on the measured values of concentrations of components, the total flow of the reaction mixture entering the reactor, and the catalyst mass *m*_cat_, as follows:1
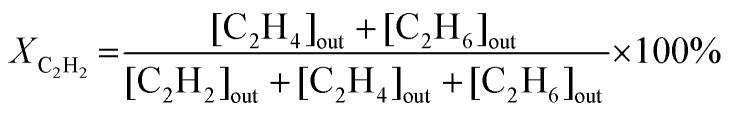
2
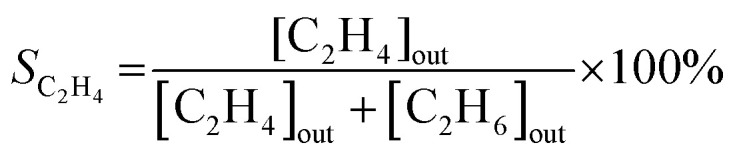
3
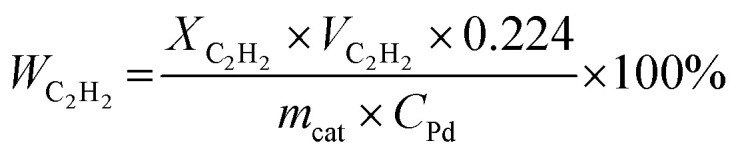
where [C_2_H_2_]_out_, [C_2_H_4_]_out_, and [C_2_H_6_]_out_ are the vol% concentrations of acetylene, ethylene and ethane at the reactor outlet, respectively, *V*_C_2_H_2__ is the C_2_H_2_ flow rate in mL s^−1^, and *C*_Pd_ is the fraction of Pd (mass%) in the catalyst sample.

Prior to the catalytic tests, all samples were pre-treated *in situ* at 250 °C in 20 mL min^−1^ flow of H_2_ for 1 h before cooling to room temperature. In a typical experiment, 20 mg of the catalyst was loaded into the reaction chamber and acetylene hydrogenation was performed at atmospheric pressure in the temperature interval of 20–85 °C and with the total feed rate varying from 5 to 200 mL min^−1^. The photocatalytic experiments were carried out in the absence of external heating, and the amount of the catalyst was reduced to 2 mg to account for the higher photocatalytic activity and limited light penetration. Single frequency, continuous wave lasers with a collimated beam of 532 nm (Cobolt Samba, HÜBNER Photonics), 633 nm (Mellet Griot), and 785 nm (IPS) were used as light sources for the wavelength-dependent photocatalytic experiments. The spot size was adjusted to approx. 2.5 mm based on the laser aperture size using an appropriate beam expander. The light intensity was controlled digitally for the Cobolt Samba laser and with neutral density filters (ThorLabs) for the other two.

The kinetic study of acetylene hydrogenation under thermal and photo-mediated activation was performed by the analysis of the initial reaction rates obtained under differential conditions. To ensure such conditions, the C_2_H_2_ conversion was kept below 10% (Fig. S1†) by varying the total flow rate at constant partial pressures of C_2_H_2_ and H_2_. To determine the apparent rate constants at different temperatures and light powers, the partial pressure of H_2_ was varied between 0.05 and 0.20 bar at a constant pressure of C_2_H_2_ of 0.05 bar. The activation energies were obtained for the temperature interval between 20 and 80 °C. For both thermal and photocatalytic experiments, the reaction temperatures were determined with an IR camera. The IR camera was calibrated by measuring the temperature of a catalyst surface uniformly heated with a hot plate and covered with CaF_2_ glass. A temperature gradient of less than 5 °C was observed between the surface temperature (IR camera) and the catalyst bottom layer (thermocouple) when the reaction temperature was controlled by an electric heater.

## Results and discussion

### Mg and Pd–Mg nanoparticles synthesis and characterization

A mixture of single crystalline platelets (mean ± standard deviation, 240 ± 50 nm) and singly twinned rod-like (310 ± 90 nm) Mg NPs (Fig. S2†), similar to previously reported mixtures,^53,54^ were obtained from the reaction of an organometallic Mg precursor with lithium naphthalenide in anhydrous conditions. The proportion of hexagonal platelets to rod-like shapes was ∼1 : 3 as measured by SEM (Fig. S2†). The polydispersity and shape heterogeneity of this Mg NPs mixture prevented shape-dependent studies and resulted in a broad extinction spectrum peaking above 800 nm (Fig. S3†), consistent with previous numerical results.^53^

The partial galvanic replacement of Mg NPs by Pd from Na_2_PdCl_4_ resulted in a layer of finely dispersed Pd, as shown in the HAADF-STEM image of Mg NPs decorated with 3 mol% Pd of Fig. 1(b) (additional images in ESI, Fig. S4†). Increasing the Na_2_PdCl_4_ concentration relative to Mg NPs produced more decorations and increased the coverage, but was accompanied by formation of larger Pd aggregates; decreasing the Na_2_PdCl_4_ concentration led to fewer Pd NPs (Fig. S5†). The Mg NP size distribution was not affected by the galvanic replacement reaction.

The bulk composition of Pd–Mg NPs measured by ICP-MS (Table 1) matched the reaction stoichiometry, as expected. The local composition was consistent with a galvanic replacement reaction: it featured small particles of Pd evenly distributed on a core of slightly oxidized Mg, with more oxidation where more Pd is present, as evidenced by STEM-EDS measurements such as those shown in Fig. 1(d), (e) and Fig. S6, 7.† A thin oxide layer is also visible on the entirety of the surface of the Mg NPs, as described in detail previously.^30,31,55^

**Table 1:** Bulk composition of Pd–Mg NPs measured by ICP-MS

Sample	Pd_added_, mol%	Pd_measured_, mol%
1 mol% Pd–Mg	1	0.94
3 mol% Pd–Mg	3	2.85
6 mol% Pd–Mg	6	5.43
9 mol% Pd–Mg	9	7.81

The increase in Pd decoration and the corresponding decrease in Mg metal content was also accompanied by a gradual decrease in optical absorbance by the Mg NPs (Fig. S3†), as expected from the loss of Mg metal. Despite the slight oxidation of Mg and the decoration with a poor plasmonic metal (Pd), the Pd–Mg NPs retained their plasmonic behavior. Indeed, a relatively simple analysis of STEM-EELS data yielded the plasmon excitation maps shown in Fig. 1(f), (g) and Fig. S7–9.† These maps show LSP excitation at the corners (low energy) and edges (high energy) of the hexagonal plates and rods, as previously reported.^30,31^ The bulk metallic plasmon of Mg, excited by an electron beam in STEM-EELS at ∼10.4 eV and mapped as shown in Fig. 1(h) and Fig. S7–9,† provides a further confirmation of the metallic character of the Mg in the NP core.

### Composition-dependent performance of bimetallic Pd–Mg catalysts

**Fig. 2:**
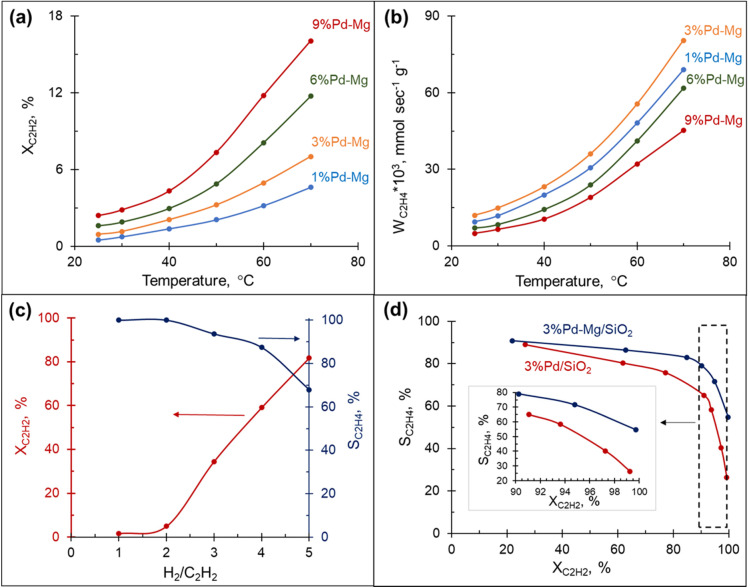
Catalytic performance of Pd–Mg bimetallic NPs supported on SiO_2_. Effect of temperature on (a) acetylene conversion and (b) ethylene formation rate, with H_2_ : C_2_H_2_ = 1 : 1. (c) Effect of hydrogen to acetylene ratio on acetylene conversion (red) and ethylene selectivity (blue) for 3%Pd–Mg/SiO_2_ at 60 °C. (d) Steady-state ethylene selectivity as a function of acetylene conversion over 3%Pd–Mg/SiO_2_ and 3%Pd/SiO_2_, with H_2_ : C_2_H_2_ = 3 : 1 at 60 °C.

The hydrogenation of acetylene proceeds efficiently over Mg NPs decorated with different amounts of Pd and supported on SiO_2_. The conversion of acetylene rapidly increased with the reaction temperature and correlated well with the amount of Pd in the sample: the highest conversion was obtained over the sample containing 9 mol% Pd and was >3.5 times higher than that obtained with the 1 mol% Pd at the same temperature (Fig. 2(a)). However, the rate of ethylene production per gram of Pd in the sample followed a different trend: it gradually increased with the decrease in Pd content from 9 to 3 mol%, then dropped down slightly with a further decrease of Pd to 1 mol% (Fig. 2(b)). This behavior is attributed to the decrease in ethylene selectivity at higher Pd content. Indeed, according to ref. 56 the presence of large Pd ensembles on the catalyst surface leads to a decrease in ethylene selectivity. The sample with 3 mol% Pd on Mg, henceforth called 3%Pd–Mg/SiO_2_, had the highest rate of ethylene production, and thus was selected for further investigation.

### Effect of H_2_ : C_2_H_2_ ratio on the catalytic performance

The amount of hydrogen in the reaction mixture had a pronounced effect on both acetylene conversion and ethylene selectivity: the conversion soared from a few percent to 80% with the increase in H_2_/C_2_H_2_ ratio from 1 to 5 (Fig. 2(c)). However, the increase in conversion was, as expected, accompanied by a decrease in ethylene selectivity. Although the low amount of hydrogen in the reaction stream limits over-hydrogenation to ethane, the catalyst could be quickly deactivated due to coke and green-oil deposition on its surface.^57^ In contrast, the hydrogen-rich stream mitigates the deactivation issue, however, the reaction must be kinetically controlled to inhibit massive over-hydrogenation and the ability of the catalyst to sustain high selectivity in a wide range of conversions becomes the key factor. The catalytic performance of 3%Pd–Mg/SiO_2_ in an excess of hydrogen was thus investigated next.

### Selectivity–conversion trends over Pd–Mg/SiO_2_*vs.* Pd/SiO_2_

To further investigate the catalytic properties of Pd–Mg NPs in selective hydrogenation, we compared the performance of two samples containing the same mass of Pd, controlled by using the same amount of Pd precursor: 3%Pd–Mg/SiO_2_ sample prepared by galvanic replacement and a monometallic Pd/SiO_2_ catalyst prepared by the conventional incipient wetness impregnation method. The 3%Pd–Mg/SiO_2_ sample demonstrated substantially higher activity in acetylene hydrogenation compared to Pd/SiO_2_ above 45 °C (Fig. S10†). An 86% acetylene conversion was observed over 3%Pd–Mg/SiO_2_ at 60 °C compared to only 20% over Pd/SiO_2_. Subsequent impregnation of Pd/SiO_2_ sample with Mg NPs did not affect its catalytic performance, confirming the absence of catalytic activity of the bare Mg NPs (Fig. S10†). The higher activity of Pd–Mg catalyst looks especially promising when comparing to the common industrial Pd–Ag catalyst, where promotion of Pd with Ag leads to a significant reduction in catalytic activity, about 20 times compared to monometallic Pd, as reported in ref. 43. A substantial decrease in selectivity with an increase in conversion is commonplace in the selective hydrogenation of unsaturated hydrocarbons. Therefore, we investigated the selectivity *vs.* conversion behavior of Pd/SiO_2_ and 3%Pd–Mg/SiO_2_ samples for an unambiguous comparison of their catalytic performance. Steady-state ethylene selectivity measured at different acetylene conversions and a constant temperature of 60 °C is shown in Fig. 2d. In these experiments, the conversion was manipulated by adjusting the total flow rate of the reagents at constant hydrogen to acetylene ratio (3 : 1). For both catalysts the ethylene selectivity gradually decreased with acetylene conversion. Over the monometallic Pd/SiO_2_ sample, the selectivity decreased from 90 to 65% with an increase of acetylene conversion from 20 to 90%, followed by a dramatic drop to ∼25% when the conversion approached 100%. Such a sharp decrease of the selectivity at high acetylene conversion over monometallic Pd catalysts is consistent with previously reported data.^37,47,48,52^ The bimetallic 3%Pd–Mg/SiO_2_ sample demonstrated substantially improved catalytic behavior. The selectivity only slightly decreased from 90 to 80% with an increase of acetylene conversion from 20 to 90%, and achieved 55% when the acetylene conversion was complete (Fig. 2d inset). In addition, the 3%Pd–Mg/SiO_2_ catalyst was stable over at least 4 h at 80 °C (Fig. S11†), during which the acetylene conversion only slightly decreased from 89 to 86% with no detectable change in ethylene selectivity.

A commonly accepted mechanism for acetylene hydrogenation includes adsorption of acetylene and dissociative adsorption of hydrogen over a metal surface followed by the successive addition of hydrogen atom for acetylene conversion to vinyl and vinyl to ethylene. The formed ethylene can be either subsequently desorbed into the gas phase, or hydrogenated further to produce undesired ethane. The effect of promotion of Pd by a second metal is generally described by electronic or geometric effects which affect energetics of Pd interaction with the surface intermidiates.^36,37,42,43,46,58^ Given we have not observed evidence of alloying between Pd and Mg in Pd–Mg nanostructures obtained by partial galvanic replacement, we hypothesize that the increased catalytic performance, in particular selectivity, in thermal experiments was due to the templating effect of Mg, leading to small well-separated Pd NPs and limiting aggregation.

### Photocatalytic properties of Pd–Mg NPs supported on glass spheres

In addition to being a competitive catalyst, the Pd–Mg platform is also an efficient visible light photocatalyst owing to the plasmonic properties of the Mg core. In order to investigate the photocatalytic performance of Pd–Mg in acetylene hydrogenation, Pd–Mg with 3 mol% Pd NPs were supported on glass spheres (GS), which increase light penetration when compared to conventional porous support materials. The resulting samples are named 3%Pd–Mg/GS.

**Fig. 3:**
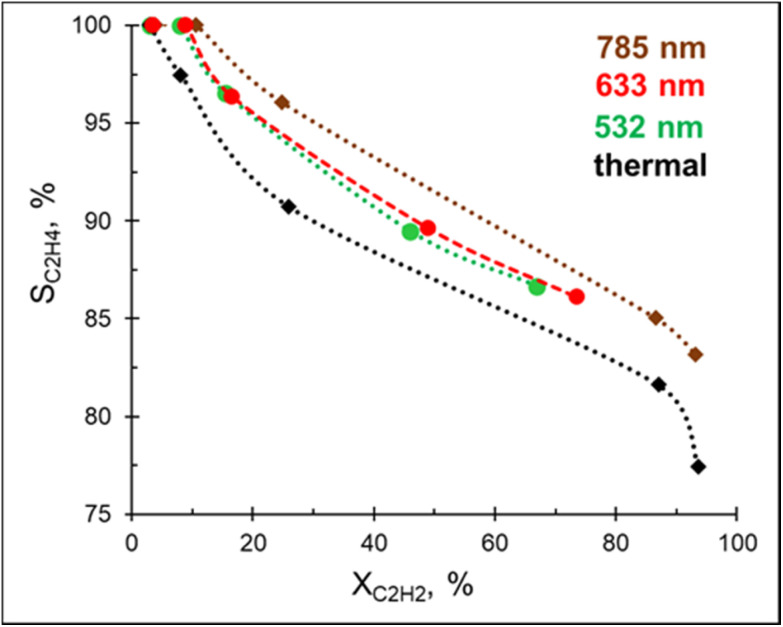
Steady-state ethylene selectivity as a function of acetylene conversion over 3%Pd–Mg/GS under thermal and photo-mediated activation. The catalyst surface temperature was maintained at 60 °C for both thermal and photocatalytic experiments and the H_2_ : C_2_H_2_ was fixed at 3 : 1.

A preliminary study revealed no hydrogenation of acetylene on bare Mg NPs supported on the glass spheres under visible light excitation, *i.e.* the bare Mg NPs are not catalytically active. Excitingly, acetylene hydrogenation efficiently proceeds under light irradiation over 3%Pd–Mg/GS in the entire investigated wavelength range (532–785 nm, Fig. 3). For both studies, the same amount of Mg NPs was used. Given that a laser beam excitation inevitably leads to photothermal heating of the catalyst surface (Fig. S12†), we chose to, for unambiguous comparison, maintain the catalyst surface temperature at 60 °C for both thermal and photocatalytic experiments. Doing so led to an acetylene conversion of >90% under a 1.8 W cm^−2^ 785 nm laser beam. The ethylene selectivity gradually decreased with acetylene conversion in both thermal and photocatalytic experiments, however, the selectivity obtained under light irradiation was higher over the entire conversion range. The highest selectivity was obtained under 785 nm laser excitation, while no significant difference was detected in selectivity between samples irradiated by 532 and 633 nm laser beams. An improvement in ethylene selectivity under white light excitation over Pd–Al NPs was previously demonstrated, where the ethylene to ethane ratio reached up to 37 (up to 10 thermally).^12^ Unfortunately, a full, direct comparison with this result is impossible as conversion–selectivity trends were not reported for the reaction catalyzed by Pd–Al NPs.

### Photo *vs.* photothermal mediated activation: kinetic analysis

The laser beam excitation of the catalyst surface is accompanied with a significant increase in the catalyst surface temperature (Fig. S12†), seemingly due to collective heating effects.^59^ The fact that these temperatures are high enough to efficiently drive the acetylene hydrogenation (Fig. 2a) together with the broad absorbance of Mg NPs (Fig. S3†) make it extremely challenging to disentangle photothermal from photochemical effects using currently established experimental procedures.^60^ Here we demonstrate that a relatively simple kinetic analysis allows to rule out purely thermally-mediated acetylene hydrogenation under a laser beam excitation over a Pd–Mg plasmonic catalyst.

**Fig. 4:**
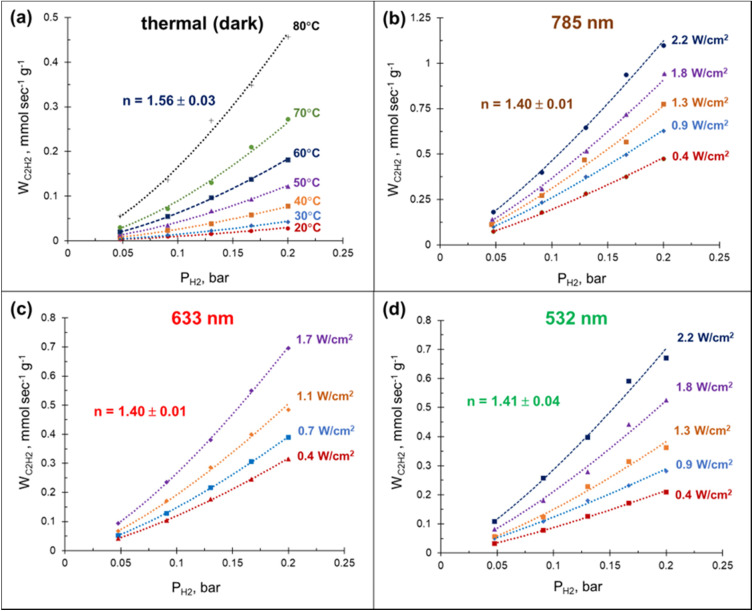
Acetylene conversion rate over 3%Pd–Mg/GS as a function of hydrogen partial pressure *P*_H_2__. (a) Thermal (dark) and photo mediated with laser light illumination wavelengths of (b) 785 nm, (c) 633 nm, and (d) 532 nm. The *P*_C_2_H_2__ was kept constant at 0.05 bar and the reaction order (*n*) reported is with respect to H_2_.

By performing acetylene hydrogenation in dark conditions at room temperature, with external heating, and under laser beam excitation without heating, we demonstrated that in both cases the ethylene formation rates increase monotonically with the hydrogen partial pressure (Fig. 4). The reaction order with respect to hydrogen, measured under dark conditions in the temperature interval of 20–80 °C was 1.56, which is slightly higher than the values 1.0–1.4 reported for other Pd-based catalysts.^39,48,61–64^ A statistically significant decrease of the reaction order to 1.4 was observed when the hydrogenation was performed under laser beam excitation in the same temperature interval, with heating provided photothermally (Fig. S12†). Although no measurable wavelength dependence of the reaction order was detected, the ethylene formation rate increased in the order 532 < 633 < 785 nm (Fig. 4(b)–(d)). The initial reaction rates under light irradiation were substantially higher compared to the dark conditions at room temperature. With a 785 nm laser light excitation (2.2 W cm^−2^), a 40 times increase in the hydrogenation rate was observed (Fig. 4(a) and (b)). Note, the direct comparison of the rates under thermal- and photo-mediated activation is hampered by our inability to measure the effective amount of catalyst involved when the reaction was performed under laser beam excitation.

**Fig. 5:**
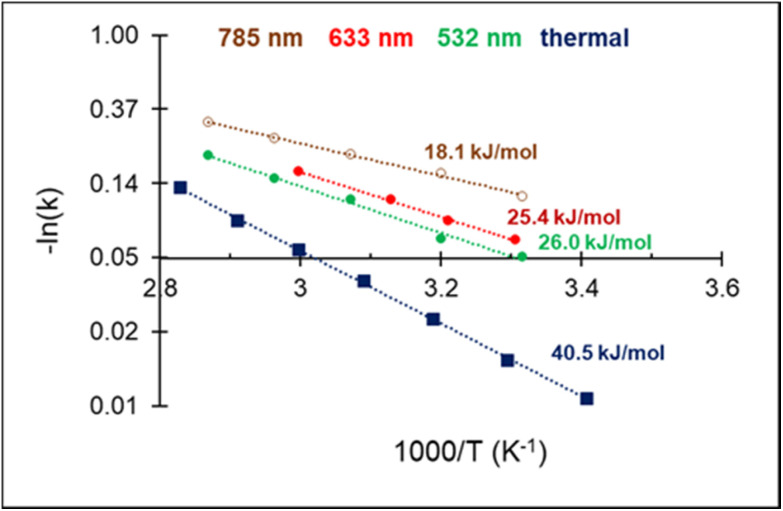
Arrhenius plot of C_2_H_2_ hydrogenation and apparent activation energies for photo- and thermocatalytic experiments over 3%Pd–Mg/GS.

The high acetylene conversion rate obtained under dark conditions implies that the reaction rate under light irradiation should be, at least in part, exponentially sensitive to the alteration in the laser power density since it is accompanied by a temperature change. In other words, the shape of the kinetic curve is defined by the ratio between photo- and thermocatalytic components in the overall reaction activation. In the extreme case of pure photothermal heating, one would obtain the same values of apparent activation energies for both photo- and thermocatalytic experiments. The temperature dependencies of the apparent rate constants of acetylene hydrogenation, extracted from the data of Fig. 4 and shown in Fig. 5, clearly demonstrate that this is not the case.

For both photo- and thermocatalytic experiments, the catalyst surface temperature was used for plotting the data in Arrhenius coordinates (Fig. 5). The apparent activation energy of 40.5 kJ mol^−1^, obtained for thermocatalytic experiments, is within the range of reported values of 30–70 kJ mol^−1^ for acetylene hydrogenation over Pd-based catalysts.^52,64–66^ A noticeable decrease in the activation energy was observed for the reaction performed under laser beam excitation. The activation energies of 26.0, 25.4, and 18.1 kJ mol^−1^ were obtained for acetylene hydrogenation under 532, 633, and 785 nm excitation, respectively (Fig. 5). The wavelength dependence of the activation energy correlates well with the change in the optical extinction of Mg NPs (Fig. S3†), where higher extinction is seen at lower energy. The observed hydrogenation rate enhancement, together with substantially lower and wavelength-dependent activation energies, provide strong evidence of the existence of different and more efficient reaction pathway(s) under light irradiation compared with thermal catalysis.

In the case of acetylene hydrogenation, however, the reaction is unlikely to proceed purely through photo-mediated activation, but rather by a photothermally driven reaction for which photo-mediated pathways are combined with a conventional thermal activation. Photothermal hydrogenation of acetylene over Pd/TiO_2_ single-atom catalyst was investigated by Guo and co-workers,^66^ who reported a substantially improved catalytic activity under white light irradiation compared to dark conditions. Their kinetic study also revealed that the apparent activation energy in photothermal catalysis was lower than in thermal catalysis. Photomediated chemical transformations can be synergistic with thermal activation, and a decrease of the apparent activation energies for thermal catalysis by illumination of a plasmonic photocatalyst has indeed been reported.^67–70^ The improved photothermal catalytic performance is usually attributed to the hot carriers-facilitated activation of reaction intermediates. However, as it was pointed out in ref. 71, photo-excited electron/hole pairs can also affect the structural parameters of the catalyst and thus improve catalytic performance. Based on XPS measurement and DFT calculations, Guo *et al.*^66^ suggested that more efficient acetylene hydrogenation under light excitation could be attributed to the facilitated activation of acetylene due to the transfer of photo-induced electrons from TiO_2_ to the adjacent Pd atoms. Meanwhile, Swearer *et al.*^12^ proposed that enhanced catalytic performance under photo-excitation was instead caused by an increased desorption rate of hydrogen from the Pd surface; this understanding has been challenged in ref. 66, where the photo-enhanced activation of hydrogen was ruled out based on H–D isotope exchange experiments. Here, we obtain relatively high reaction orders with respect to hydrogen, which could suggest a H_2_-starved Pd surface, however, the more positive reaction order we obtained under dark conditions (Fig. 4) is in clear contradiction with the mechanism invoking increased desorption of hydrogen under light excitation.

## Conclusion

We demonstrated that the coupling of the plasmonic properties of earth-abundant Mg with the high catalytic activity of Pd is an efficient strategy to manipulate the performance of industrially relevant selective hydrogenation processes. Mg NPs decorated with finely dispersed Pd, 1 to 9 mol% relative to Mg, were synthesized by partial galvanic replacement. The resulting structures retained their metallic and plasmonic character, as shown by STEM-EELS and optical extinction spectroscopy.

Pd–Mg bimetallic nanocomposites were shown to be promising highly selective hydrogenation catalysts under conventional thermally-driven conditions. Pd–Mg samples demonstrated substantially improved catalytic behavior compared to Pd alone: an ethylene selectivity of 55% was achieved at complete acetylene conversion in an excess of hydrogen. This improved catalytic behavior was attributed to the small well-separated Pd NPs obtained on Mg due to the surface's ability to limit Pd aggregation.

Enhanced activity and selectivity in acetylene hydrogenation were observed under light excitation compared to the thermally activated process under dark conditions. Kinetic analysis revealed a substantially lower and wavelength-dependent activation energy of photo-induced hydrogenation, providing strong evidence for a plasmonically affected catalytic behavior. This study and the use of earth-abundant plasmonics in photocatalysis paves the way for more resource-efficient, green approaches to industrially-relevant chemical reactions.

## Author contributions

E. R. and V. L. designed and planned the experiments. V. L. performed catalytic and galvanic replacement experiments and wrote the manuscript draft, T. M. R. W. and E. R. H. synthesized Mg nanoparticles, E. R., Y. I. and G. D. performed electron microscopy. All authors contributed to editing the manuscript.

## Conflicts of interest

There are no conflicts of interest to declare.

## Supplementary Material

NR-015-D3NR00745F-s001
