# Supplementary material for: Plasmonic magnesium nanoparticles decorated with palladium catalyze thermal and light-driven hydrogenation of acetylene
Source: Nanoscale. 2023 Mar 29;15(16):7420–9. doi: 10.1039/d3nr00745f (PMC10134437; doi:10.1039/d3nr00745f)
Supplement: NR-015-D3NR00745F-s001 [file NR-015-D3NR00745F-s001.pdf]

Electronic Supplementary Material (ESI) for Nanoscale.
This journal is © The Royal Society of Chemistry 2023

Supporting Information for Plasmonic Magnesium Nanoparticles Decorated with Palladium Catalyze Thermal and Light-driven Hydrogenation of Acetylene

Vladimir Lomonosov,[1,2] Thomas M.R. Wayman,[1,2] Elizabeth R. Hopper,[1,2,3] Yurii P. Ivanov,[4] Giorgio Divitini,[4] and Emilie Ringe[1,2]*

1. Department of Materials Science and Metallurgy, University of Cambridge, 27 Charles Babbage Road, Cambridge, United Kingdom, CB3 0FS, United Kingdom
2. Department of Earth Sciences, University of Cambridge, Downing Street, Cambridge, United Kingdom, CB2 3EQ, United Kingdom
3. Department of Chemical Engineering and Biotechnology, University of Cambridge, Philippa Fawcett Drive, Cambridge CB3 0AS, United Kingdom
4. Electron Spectroscopy and Nanoscopy, Istituto Italiano di Tecnologia, Via Morego 30, 16163 Genova, Italy

* Corresponding author: er407@cam.ac.uk; +44 (0)1223 334330 (ph.), +44 (0)1223 334567 (fax).

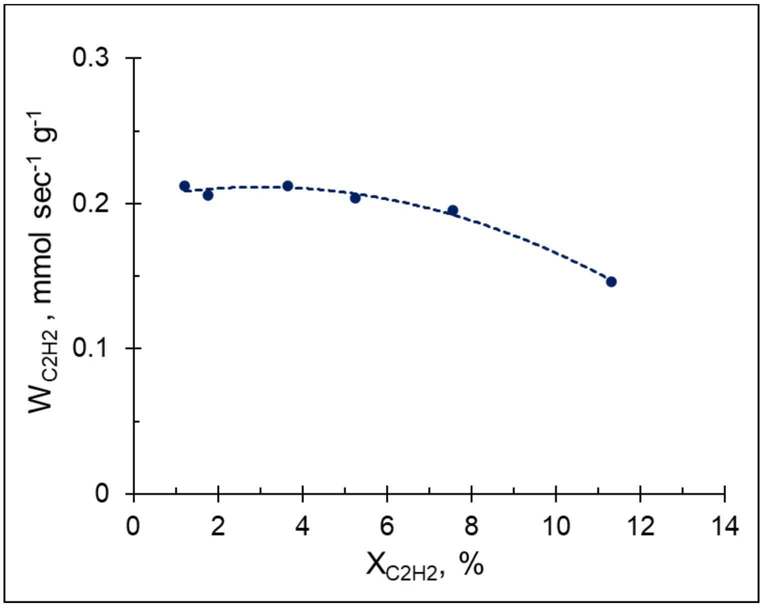

Figure S1. Acetylene conversion rate as a function of acetylene conversion over 3%Pd-Mg supported on glass spheres (GS); T = 60°C, H_2:C_2H_2 = 5:1.

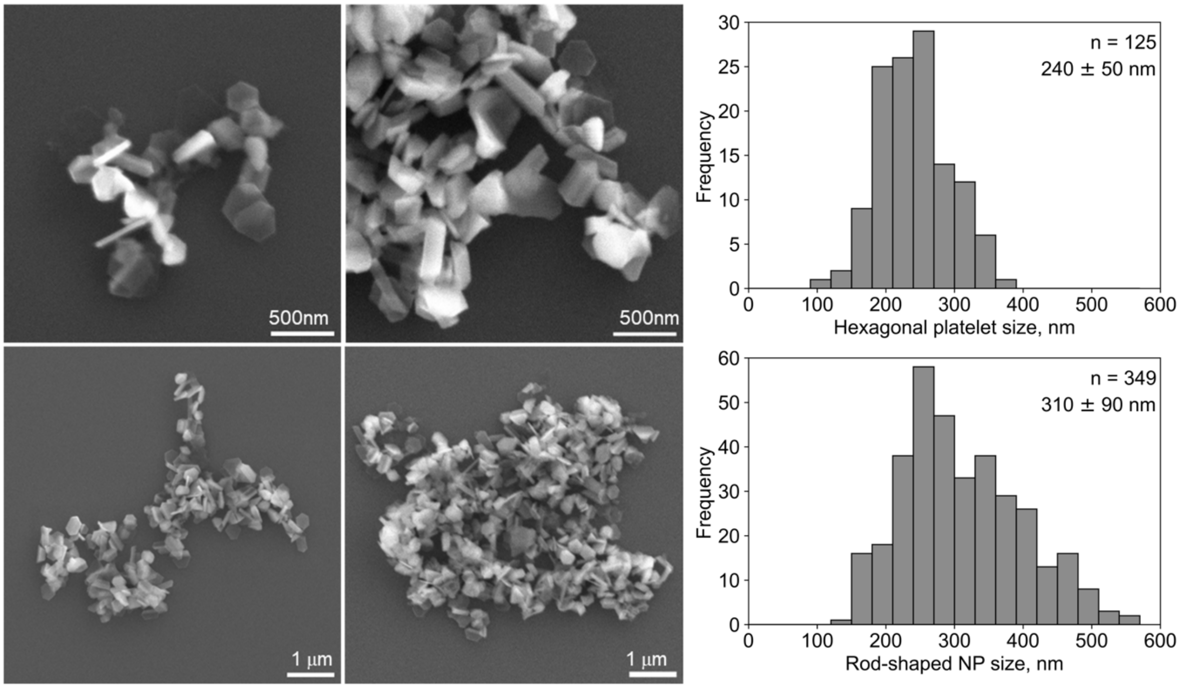

Figure S2. Representative SEM images and size distributions of hexagonal platelets and rod-shaped Mg NPs before partial galvanic replacement. On the histograms, n represents the number of NPs measured and the mean is reported together with the standard deviation.

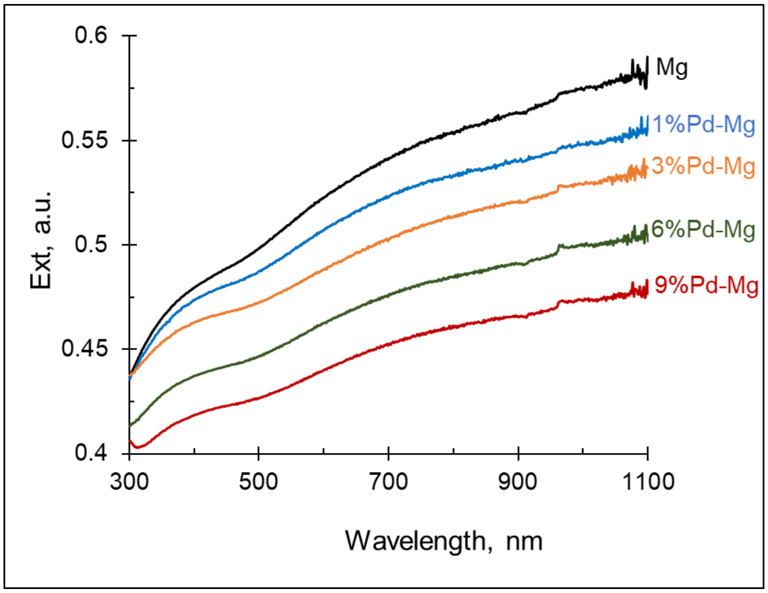

Figure S3. Extinction spectra of Mg NPs (black) and Mg NPs decorated with different mol% of Pd, suspended in isopropanol.

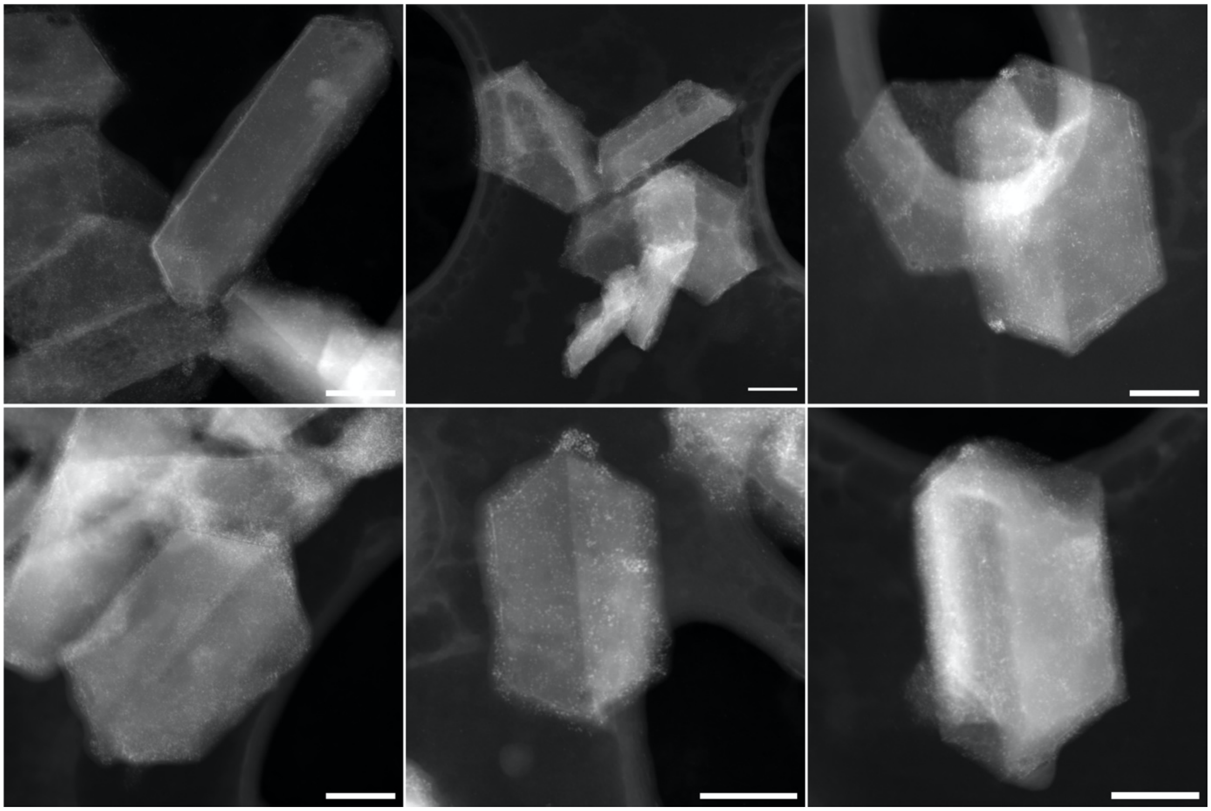

Figure S4. Additional representative STEM-HAADF images of Mg NPs decorated with 3 mol% Pd. Scale bars, 100 nm.

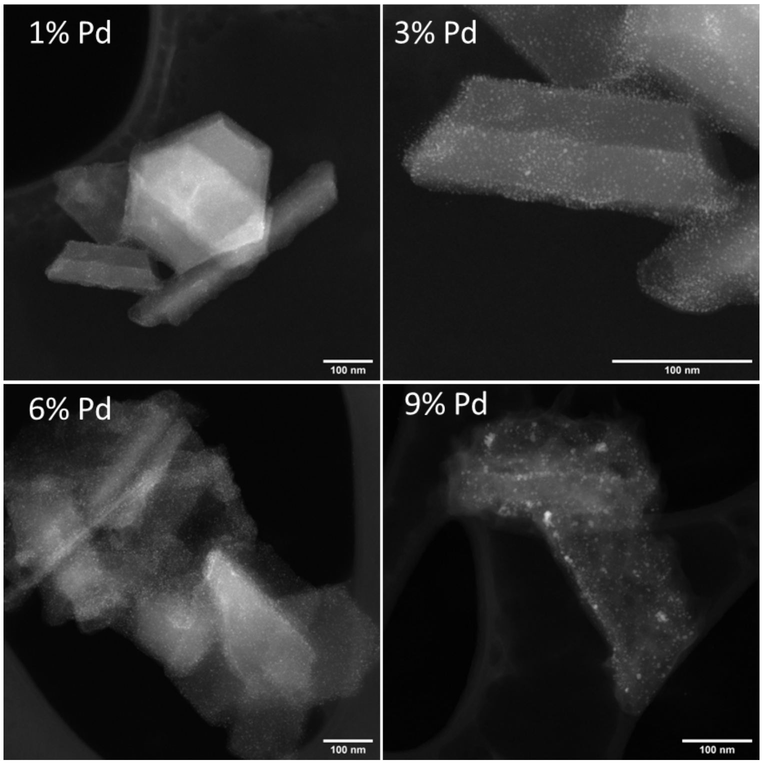

Figure S5. Representative HAADF-STEM images of Mg NPs decorated with different mol% of Pd.

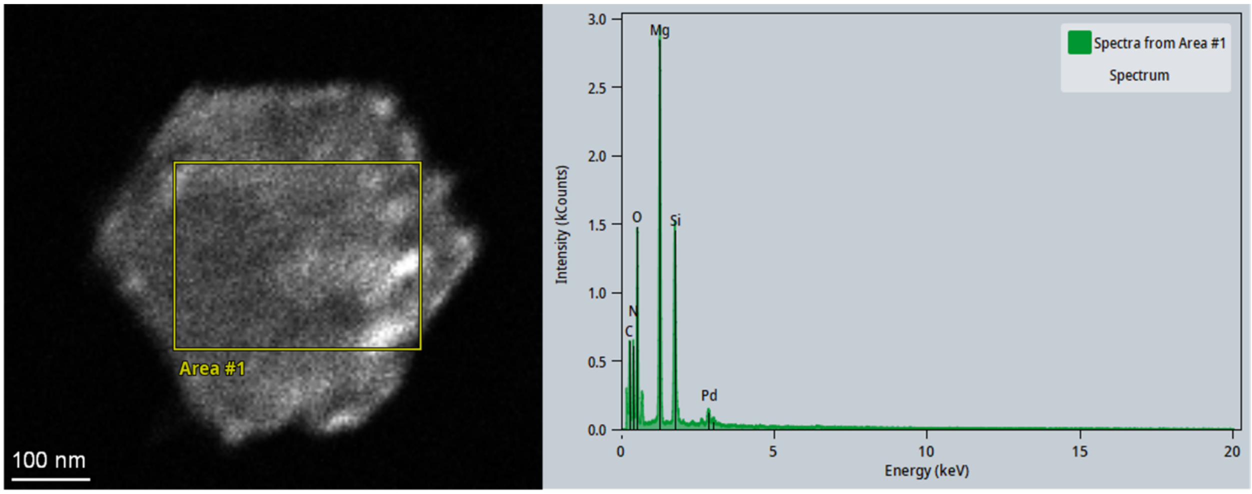

Figure S6. HAADF-STEM images acquired concurrently to the STEM-EDS data presented in Figure 1d-e and integrated spectrum in the area defined on the image, showing Mg, O, Pd, and C from the sample and Si, N from the support membrane.

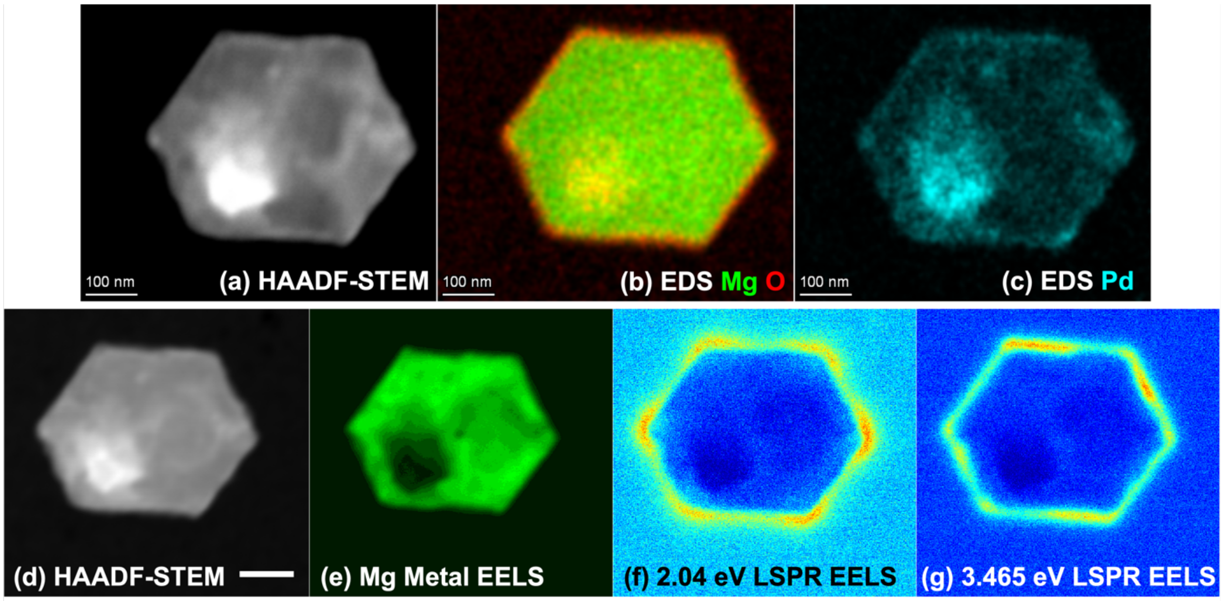

Figure S7. Additional STEM-EDS and STEM-EELS data on the same NP confirming the presence of a thin oxide layer and the metallic, plasmonic nature of Pd-decorated Mg NPs (3 mol% Pd). (a) HAADF-STEM acquired concurrently to the STEM-EDS maps, (b,c) STEM-EDS maps showing the spatial distribution of Mg, O, and Pd, (d) HAADF-STEM acquired concurrently to the STEM-EELS maps, and (e-g) STEM-EELS intensity integrated in a 0.105 eV window centred at 10.395 eV, 2.04 eV, and 3.465 eV, respectively. The scale bar in (d) is 100 nm and applies to (e-g).

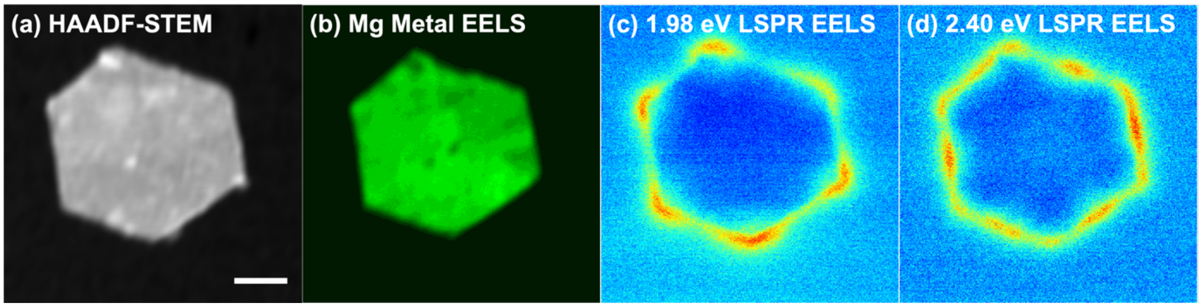

Figure S8. Additional STEM-EELS data supporting the metallic, plasmonic nature of hexagonal Pd-decorated Mg NPs (3 mol% Pd). (a) HAADF-STEM, and (b-d) STEM-EELS intensity integrated in a 0.105 eV window centred at 10.335 eV, 1.98 eV, and 2.40 eV, respectively. The scale bar in (a) is 100 nm and applies to all panels.

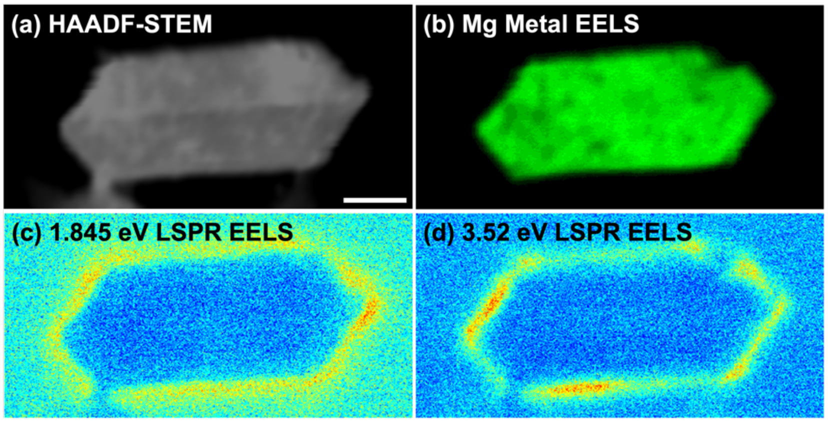

Figure S9. Additional STEM-EELS data supporting the metallic, plasmonic nature of rod-shaped Pd-decorated Mg NPs (3 mol% Pd). (a) HAADF-STEM, and (b-d) STEM-EELS intensity integrated in a 0.105 eV window centred at 10.455 eV, 1.845 eV, and 3.52 eV, respectively. The scale bar in (a) is 100 nm and applies to all panels.

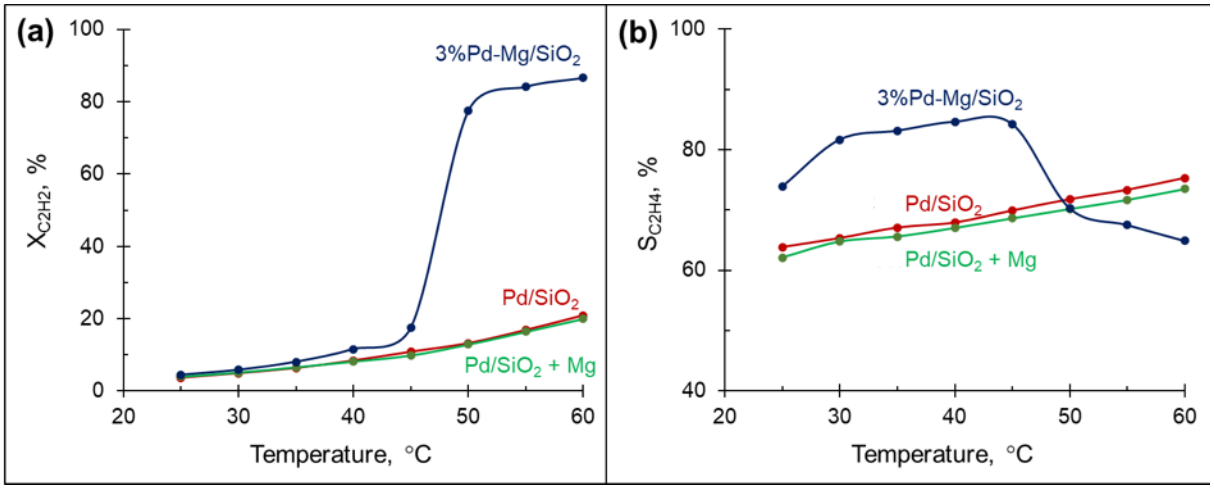

Figure S10. Effect of temperature on (a) acetylene conversion and (b) ethylene selectivity over Pd/SiO_2, Pd/SiO_2 + Mg NPs, where the Pd and Mg are not synthesized together, and the galvanic replacement-produced 3%Pd-Mg/SiO_2. H_2:C_2H_2 = 3:1.

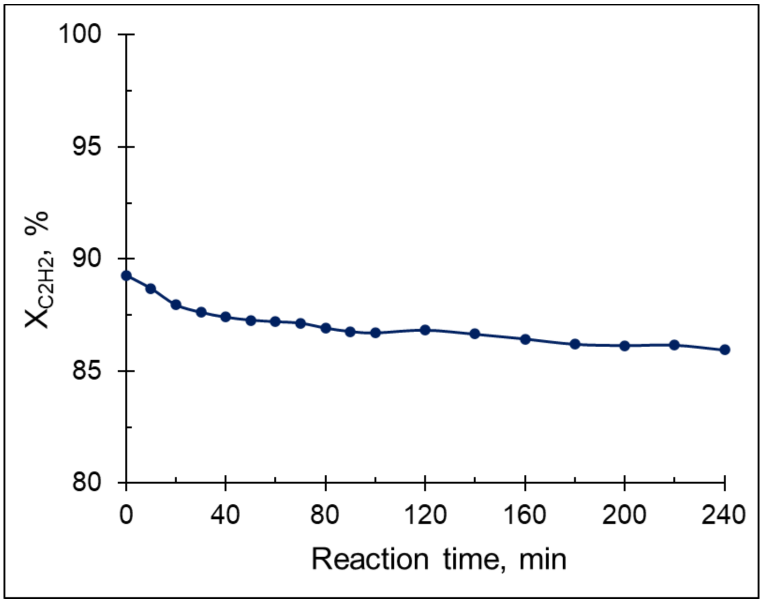

Figure S11. Stability of 3%Pd-Mg/SiO_2 at 80°C, H_2:C_2H_2 = 3:1.

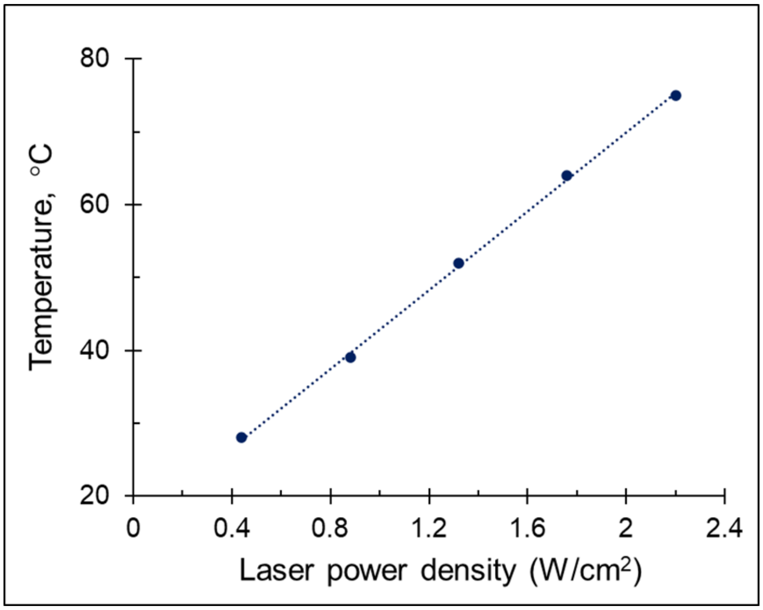

Figure S12. Surface temperature of 3%Pd-Mg/GS measured by an IR camera as a function of the 532 nm laser power density.
